# *Prevotella* genus and its related NOD-like receptor signaling pathway in young males with stage III periodontitis

**DOI:** 10.3389/fmicb.2022.1049525

**Published:** 2022-12-08

**Authors:** Yaqiong Zhao, Qin Ye, Yao Feng, Yun Chen, Li Tan, Zeyue Ouyang, Jie Zhao, Jing Hu, Ningxin Chen, Xiaolin Su, Marie Aimee Dusenge, Yunzhi Feng, Yue Guo

**Affiliations:** Department of Stomatology, The Second Xiangya Hospital, Central South University, Changsha, Hunan, China

**Keywords:** periodontitis, *Prevotella intermedia*, NLR proteins, oral microbiota, 16S rRNA sequence analysis

## Abstract

**Background:**

As periodontitis progresses, the oral microbiota community changes dynamically. In this study, we evaluated the dominant bacteria and their roles in the potential pathway in young males with stage III periodontitis.

**Methods:**

16S rRNA sequencing was performed to evaluate variations in the composition of oral bacteria between males with stage I and III periodontitis and identify the dominant bacteria of each group. Function prediction was obtained based on 16S rRNA sequencing data. The inhibitor of the predominant pathway for stage III periodontitis was used to investigate the role of the dominant bacteria in periodontitis *in vivo* and *in vitro*.

**Results:**

Chao1 index, Observed Species and Phylogenetic Diversity (PD) whole tree values were significantly higher in the stage III periodontitis group. β-diversity suggested that samples could be divided according to the stages of periodontitis. The dominant bacteria in stage III periodontitis were *Prevotella*, *Prevotella_7*, and *Dialister*, whereas that in stage I periodontitis was *Cardiobacterium*. KEGG analysis predicted that variations in the oral microbiome may be related to the NOD-like receptor signaling pathway. The inhibitor of this pathway, NOD-IN-1, decreased *P*. *intermedia* -induced *Tnf-α* mRNA expression and increased *P*. *intermedia* -induced *Il-6* mRNA expression, consistent with the ELISA results. Immunohistochemistry confirmed the down-regulation of TNF-α and IL-6 expressions by NOD-IN-1 in *P*. *intermedia*–induced periodontitis.

**Conclusion:**

The composition of the oral bacteria in young males varied according to the stage of periodontitis. The species richness of oral microtia was greater in young males with stage III periodontitis than those with stage I periodontitis. *Prevotella* was the dominant bacteria in young males with stage III periodontitis, and inhibition of the NOD-like receptor signaling pathway can decrease the periodontal inflammation induced by *P*. *intermedia*.

## Introduction

Periodontitis is a chronic inflammatory infectious disease characterized by periodontal ligament tissue destruction, alveolar bone resorption, and tooth loss (Pihlstrom et al., 2005). In the fourth national oral health survey in mainland China, the prevalence of adults with periodontitis was 52.8%, and the prevalence of severe periodontitis (stage III or IV) was 10.6%, which places a serious burden on society (Jiao et al., 2021). Thus, prevention and treatment of the disease are very important.

The host immunological responses to periodontitis microorganisms play a crucial role in disease progression. Innate immunity is the first line of defense and has a vital role in defending against multiple microbial pathogens (Cekici et al., 2000). The innate immune cells recognize pathogen-derived molecular molecules by expressing a family of receptors known as pattern recognition receptors (PRRs) (Brubaker et al., 2015). Nucleotide-binding oligomerization domain-containing protein 1 (NOD1) and NOD2 are the most-studied in the nucleotide-binding domain and leucine-rich repeat-containing receptor (NLR) family that sense bacterial cell wall components (Di Virgilio, 2013). Studies have shown that lipopolysaccharide (LPS) can upregulate the expression levels of NOD1 and NOD2 (Mulla et al., 2009). NOD agonists can synergistically increase the expression of LPS-induced pro-inflammatory mediators, such as TNF-α, IL-1β, and IL-6 (Tsai et al., 2011).

Young adults have a more efficient immuno-inflammatory response than old people (Ebersole et al., 2000), but the role of NOD in the process remains unclear. In periodontitis, with aggravation of the disease, the bacterial composition changes, along with the immune inflammatory response (Nibali et al., 2022). Current studies have been unable to clarify whether NOD plays a pro-inflammatory or anti-inflammatory role in periodontitis. It has been reported that knockout of NOD1/NOD2 promotes alveolar bone resorption (Prates et al., 2014; Chaves de Souza et al., 2016). However, other scholars have found that NOD1 and NOD2 protect periodontal tissue and attenuate periodontal inflammation and alveolar bone resorption (Jiao et al., 2013; Souza et al., 2016).

This study sought to evaluate the subgingival bacterial microbiome in young males with different stages of periodontitis and analyze the signaling pathways through which the dominant bacteria of stage III periodontitis play a role.

## Materials and methods

### Subject population and inclusion criteria

The Ethics Committee of the Second Xiangya Hospital of Central South University provided ethics approval. All participants fully understood the research content and signed an informed consent form. The clinical trial registration number is ChiCTR2100046828. We enrolled a total of 31 participants who visited the Stomatology Medicine Center of the Second Xiangya Hospital of Central South University during November 19, 2020, and January 19, 2021. Male patients were selected if they were aged 20–44 years and had ≥15 natural teeth. Exclusion criteria included: periodontal treatment in the last 6 months, smoking, cancer, systemic disease, the use of antibiotics in the past 30 days, or any other oral disease. All participants underwent a comprehensive and detailed oral examination to assess the presence and severity of clinical periodontal disease based on the standard of the 2017 World Workshop on the Classification of Periodontal and Peri-Implant Diseases and Conditions (Papapanou et al., 2018). Disease severity was recorded based on the clinical attachment level (CAL) and the number of missing teeth as follows: CAL of 1–2 mm (stage I), 3–4 mm (stage II), or ≥ 5 mm (stage III). Participants with ≤4 teeth missing were considered stage III cases, while those with ≥5 teeth missing were considered stage IV cases. Patients with stage III and stage IV periodontitis were combined into a stage III group.

### Subgingival plaque collection

Subgingival plaques were obtained using a sterile Gracey scraper from the bottom of the periodontal pocket. The samples were transferred to 1.5 mL Eppendorf microcentrifuge tubes containing 1 mL of phosphate-buffered saline (PBS) and frozen immediately at −80°C.

### DNA extraction and sequencing

According to the manufacturer’s instructions, metagenomic DNA was isolated using the DNeasy PowerSoil kit (Qiagen, Hilden, Germany). DNA was quantified using a NanoDrop 2000 spectrometer (Thermo Fisher Scientific, Waltham, MA, United States), and the samples were loaded into the 1% agarose gel pore and subjected to 120 V constant pressure electrophoresis for 15 min. Sequencing of 16S rRNA was performed by OEBiotech (Shanghai, China). The sequencing procedures were carried out as previously reported (Zhao et al., 2021a). Briefly, the metagenomic DNA was used as template for 16S rRNA amplification with the following primers: 343 Forward (5’-TACGGRAGGCAGCAG-3′); 798 Reverse (5’-AGGGTATCTAATCCT-3′). Amplicon quality was purified with AMPure XP beads (Agencourt, Beckman, United States), and amplified for another round of PCR. After purified with the AMPure XP beads again, the final amplicon was quantified using Qubit dsDNA assay kit (Life Technologies, California, United States). Equal amounts of purified amplicon were pooled for subsequent sequencing.Illumina MiSeq (V1.9.1) was used to generates the raw reads, and the sequences were processed and analyzed through Trimmomatic (V.0.35), Flash (V 1.2.11), QIIME (V 1.8.0), and UCHIME (V 2.4.2).

### Bioinformatics analysis

Microbial diversity was evaluated using α-diversity and β-diversity. The α-diversity indices included Chao1, Good’s coverage, PD whole tree, the Shannon index, and the Simpson index. The β-diversity was assessed using principal component analysis (PCA), principal coordinate analysis (PCoA), and the non-metric multidimensional scale (NMDS) to plot the similarities or differences of samples’ community composition. Microbial community metagenomic prediction was carried out using PICRUSt (Langille et al., 2013) based on the GreenGenes database. Differential taxa analyses were performed using the linear discriminant analysis effect size (LEfSe) (Chang et al., 2022) Function enrichment was identified using Kyoto Encyclopedia of Gene and Genomes (KEGG) pathways.

### LPS isolation

As described previously (Choi et al., 2014), LPS was prepared from freeze-dried *P*. *intermedia* cells using the conventional hot phenol-water procedure. *P*. *intermedia* ATCC25611 was cultured on blood AGAR plates, and the strain was scraped off and resuspended with normal saline to a volume of 100 mL after centrifugation at 4,000 rpm for 20 min at 4°C. After repeated freezing and thawing for three time, the bacterial suspension was heated together with the same amount of 90% phenol at 68°C for 30 min. The aqueous solution was collected and dialyzed for 12 h to remove the phenol, and then dialyzed for 60 h in distilled water. The crude LPS was concentrated with 50% polyethylene glycol. DNase (50 μg/mL) and RNase (50 μg/mL) were added at 37°C for 4 h, then heated in 100°C for 10 min. After centrifugation at 1500 r/min for 30 min, discard the precipitate, acetone was added to the supernatant, purified LPS was obtained after precipitation. The contaminating proteins and nucleic acids were removed using proteinase K, DNase, and RNase. We trusted the Shanghai Bioresource Collection Center (Shanghai, China) to complete the purification of LPS.

### Cell cultivations

RAW264.7 murine macrophages were cultured in Dulbecco’s modified Eagle medium supplemented with 10% heat-inactivated fetal bovine serum, 100 U/mL of penicillin, and 100 μg/mL of streptomycin in a humidified chamber with 5% CO_2_ at 37°C. The cells were incubated in six-well culture plates at a density of 2 × 10^6^ cells/well for 24 h to adhere to the plates. Cells were then treated with 10 μL/mL of P. intermedia LPS in the absence or presence of different doses of NOD-IN-1 for another 24 h. After culturation, RNA and protein were collected for further investigation.

### RNA preparation and real-time polymerase chain reaction

cDNA was amplified utilizing a TB Green® Premix Ex Taq™ II kit (Takara Bio Inc., Shiga, Japan) and the LightCycler® 96 system (Roche, Basel, Switzerland). The amplification was conducted for 40 cycles of 95°C for 5 s and 60°C for 20 s. The oligonucleotide primers were listed in Table 1.

**Table 1:** Nucleotide sequence of primers used for RT-PCR.

Target		Primers (5′-3′)
Il-6	Forward	CTCCCAACAGACCTGTCTATAC
	Reverse	CCATTGCACAACTCTTTTCTCA
Tnf-α	Forward	ATGTCTCAGCCTCTTCTCATTC
	Reverse	GCTTGTCACTCGAATTTTGAGA
β-actin	Forward	TGAGAGGGAAATCGTGCGTGCGTGAC
	Reverse	GCTCGTTGCCAATAGTGATGACC

### Western blot analysis

After culturation as described above, the cells were washed with PBS twice, and protein was extracted using lysis buffer. Twenty micrograms of the total protein were loaded and electrophoresed on a 12% (w/w) sodium dodecyl sulfate–polyacrylamide gel after being quantified with a BCA kit (Beyotime, Shanghai, China). The resolved proteins were blotted onto a nitrocellulose membrane, and the membranes were then incubated with the primary antibody hepcidin (1:1000, Abcam, Cambridge, United Kingdom), followed by treatment with the corresponding secondary antibodies. The immunoreactive bands were visualized with NcmECL ultra-reagent (NCM Biotech, Suzhou, China). Densitometry of protein bands was performed using ImageJ (U.S. National Institutes of Health, Bethesda, MD, United States).

### Enzyme-linked immunosorbent assay

IL-6 and TNF-α concentrations in the cell supernatant were measured with corresponding ELISA kits (Invitrogen, CA, United States). The OD value of each well was measured at 450 nm using a spectrophotometer, and the concentration was calculated according to the standard curve of IL-6 and TNF-α.

### Animals

Male C57BL/6 mice (8 weeks old) were purchased from SJA Laboratory Animal Co., Ltd. (Changsha, China) and housed under specific pathogen-free conditions with free access to food and tap water. The animal experiment protocols were approved by the Experimental Animal Ethics Committee of the Second Xiangya Hospital. The mice were divided into an LPS group, LPS + DMSO) group, LPS + NOD-IN-1 group, and control group. NOD-IN-1 and its vehicle, 1%DMSO, were administered by intraperitoneal injection before 2 h of gingival injection of LPS. The experimental group mice received *P*. *intermedia* LPS injections using a Hamilton 1700 syringe (Hamilton, Bonadoz, Switzerland); specifically, 5 mg/μL of LPS was injected into the gingiva around the mandibular first molar every 48 h for a total of 13 times. In the control group, PBS was given instead of LPS. To facilitate LPS injection, anesthesia was induced with 1% pentobarbital sodium. At the end of the injection period, the animals were sacrificed by cervical dislocation, and mandibular specimens were dissected, fixed in 4% paraformaldehyde phosphate buffer solution for 48 h, and decalcified in 17% for 21 days.

### Histological analysis

The mandibles were embedded in paraffin, and 4-μm-thick mesiodistal sections were prepared. The sections exhibiting the entire molar roots were selected for HE staining and observed under a light microscope (Leica Microsystems, Wetzlar, Germany). The distance of the cementoenamel junction–alveolar bone crest (CEJ-ABC) was calculated. Immunohistochemical staining for TNF-α and IL-6 was performed. Deparaffinized and ethanol-rehydrated sections were incubated with 0.25% pancreatic enzyme at 37°C for 30 min and treated with 3% H_2_O_2_ in the dark for 15 min. After being blocked with 5% solution for 30 min, the sections were incubated with the following primary antibodies overnight at 4°C: anti-mouse TNF-α (Abcam, Cambridge, United Kingdom), anti-rabbit IL-6 (Proteintech Corp., Madison, WI, United States), and anti-mouse hepcidin (Proteintech Corp., Madison, WI, United States). Then, the sections were incubated with diaminobenzidine (DAB) solution and counterstained with hematoxylin. After gradient dehydration, the sections were mounted with neutral gum and observed under an optical microscope (Leica Microsystems, Wetzlar, Germany).

### Statistical analysis

Experiment data are presented as mean ± standard deviation values. One-way analysis of variance was performed to judge the significant differences within the groups. The comparison of two groups was evaluated by Tukey’s post-hoc comparisons. *p* < 0.05 was considered to be statistically significant.

## Results

### Characteristics of selected patients

The mean ± standard deviation ages were 33.75 ± 5.88 years and 35.73 ± 5.93 years for young males with stage I and III periodontitis, respectively. There was no statistical difference in age between the two groups (*p* > 0.05).

### *Prevotella* was most abundant in young males with stage III periodontitis

**Figure 1:**
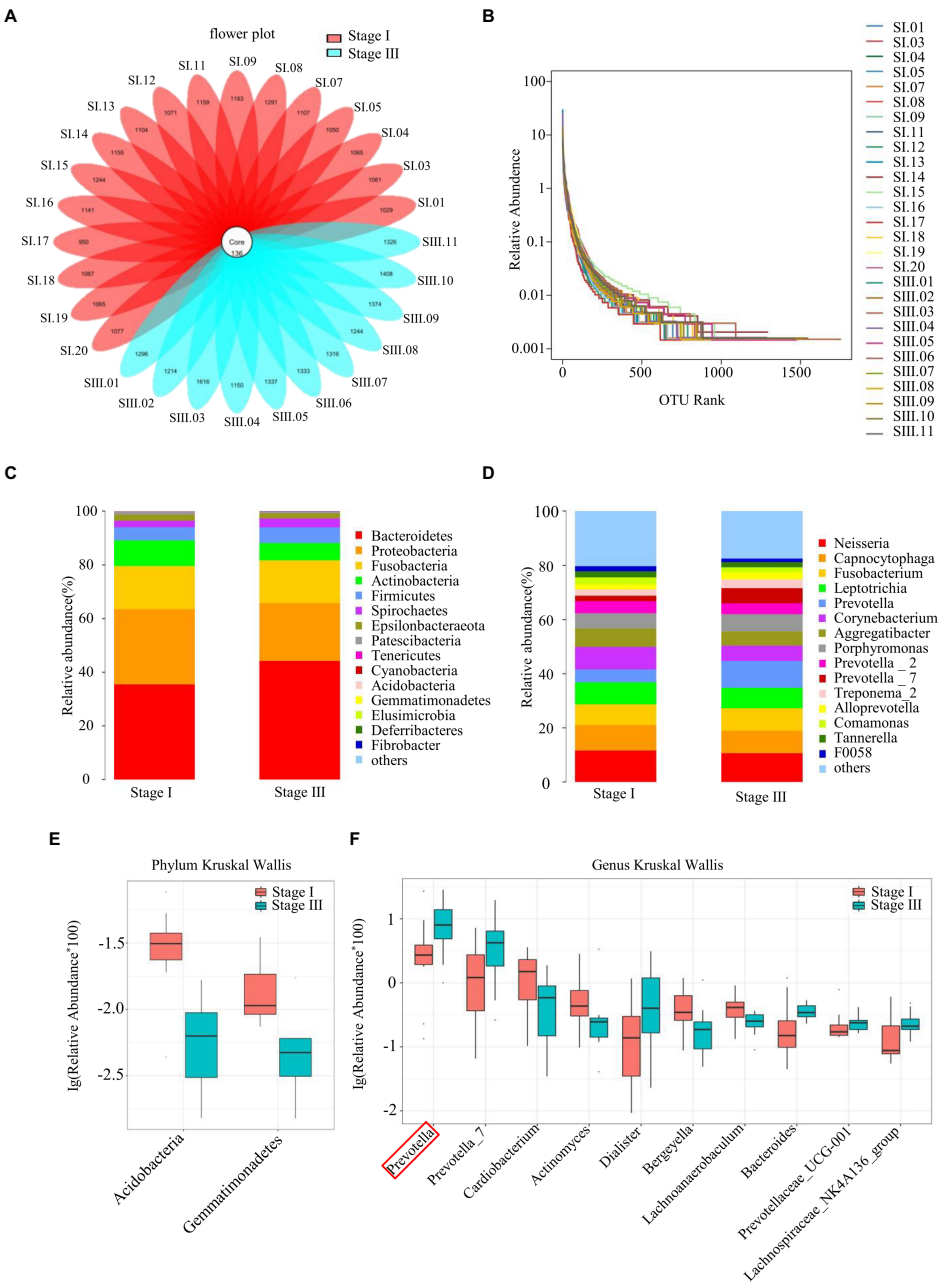
Sequence quality and composition of oral microbiomes. **(A)** A flower plot. **(B)** Rank–abundance curves for all samples. Comparison of the composition of oral microbiomes between stage I and stage III samples at the phylum **(C)** and genus **(D)** levels. Analysis of species differences between stage I and stage III samples by the Kruskal–Wallis test at the phylum **(E)** and genus **(F)** levels.

The flower diagram showed that there were 136 common OTUs in all patients (Figure 1A). The sequencing quality was assessed by rank–abundance curves, and there was no significant difference in OTU numbers between patients (Figure 1B). The quality of DNA sample extraction was determined by agarose gel electrophoresis (Supplementary Figure S1). Although the oral microbial composition of the two groups was similar, the abundance of each species was quite different (Figures 1C,D). Kruskal–Wallis tests were performed to evaluate the species differences between the two groups at the phylum and genus levels (Figures 1E,F). At the phylum level, Acidobacteria and Gemmatimonadetes were less common in the stage III group than the stage I group. Compared to the stage I group, the relative abundance of *Prevotella* increased by 2.11-fold, *Prevotella 7 by* 2.82-fold, *Dialister* by 3.29-foldin the stage III group, whereas the relative abundance of *Cardiobacterium* decreased by 2.44-fold, *Actinomyces* by 1.39-fold, and *Bergeyella* by 1.68-fold (Supplementary Table S1).

### *Prevotella* was a potential biomarker in young males with stage III periodontitis who had a greater richness of oral bacteria

**Figure 2:**
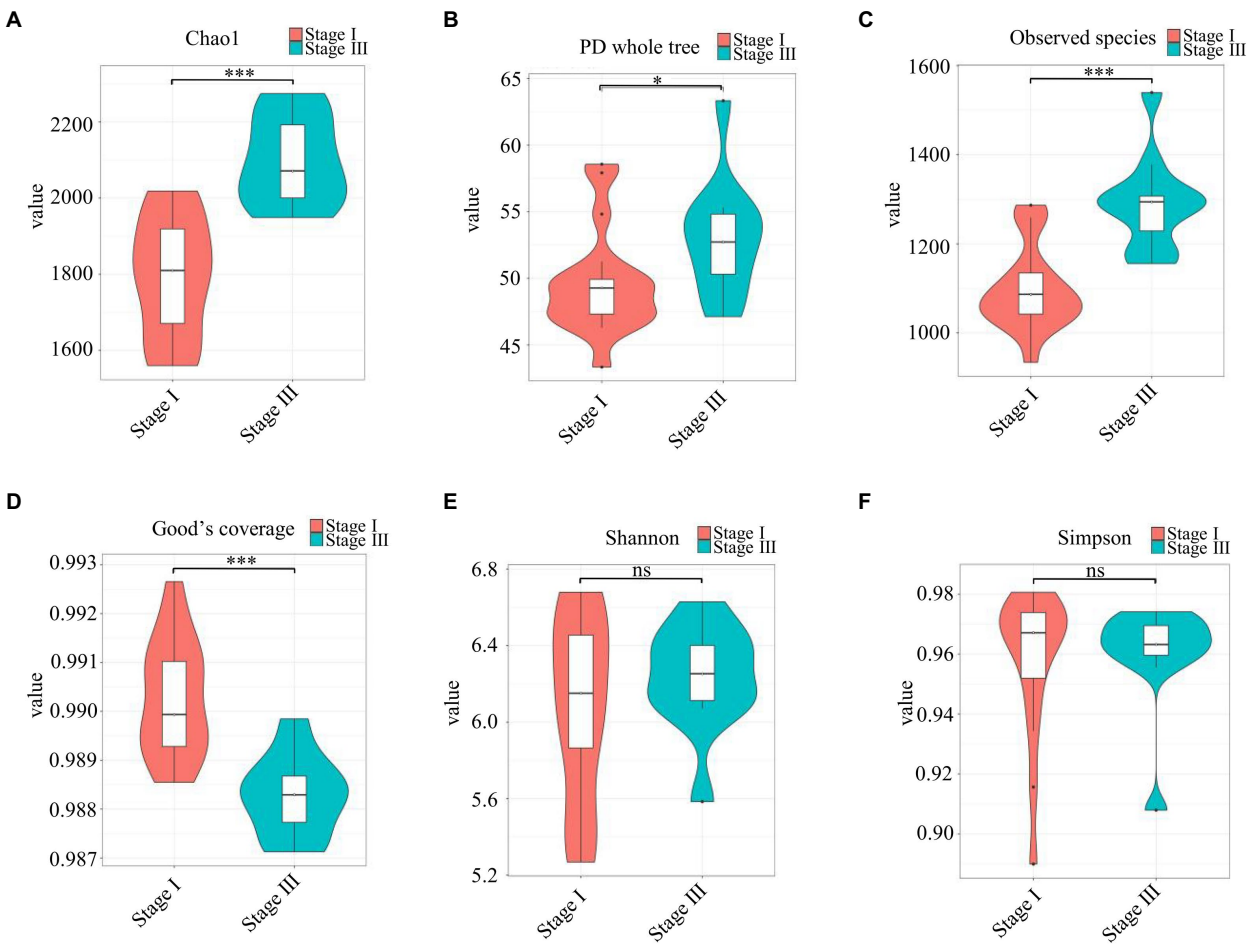
The α-diversity analysis. **(A–F)** Violin plots of α-diversity indices (Chao1, Good’s coverage, observed species, PD whole tree, Simpson, and Shannon, respectively) comparing stage I (red) and stage III (green) samples. (*p < 0.01, ***p < 0.001).

**Figure 3:**
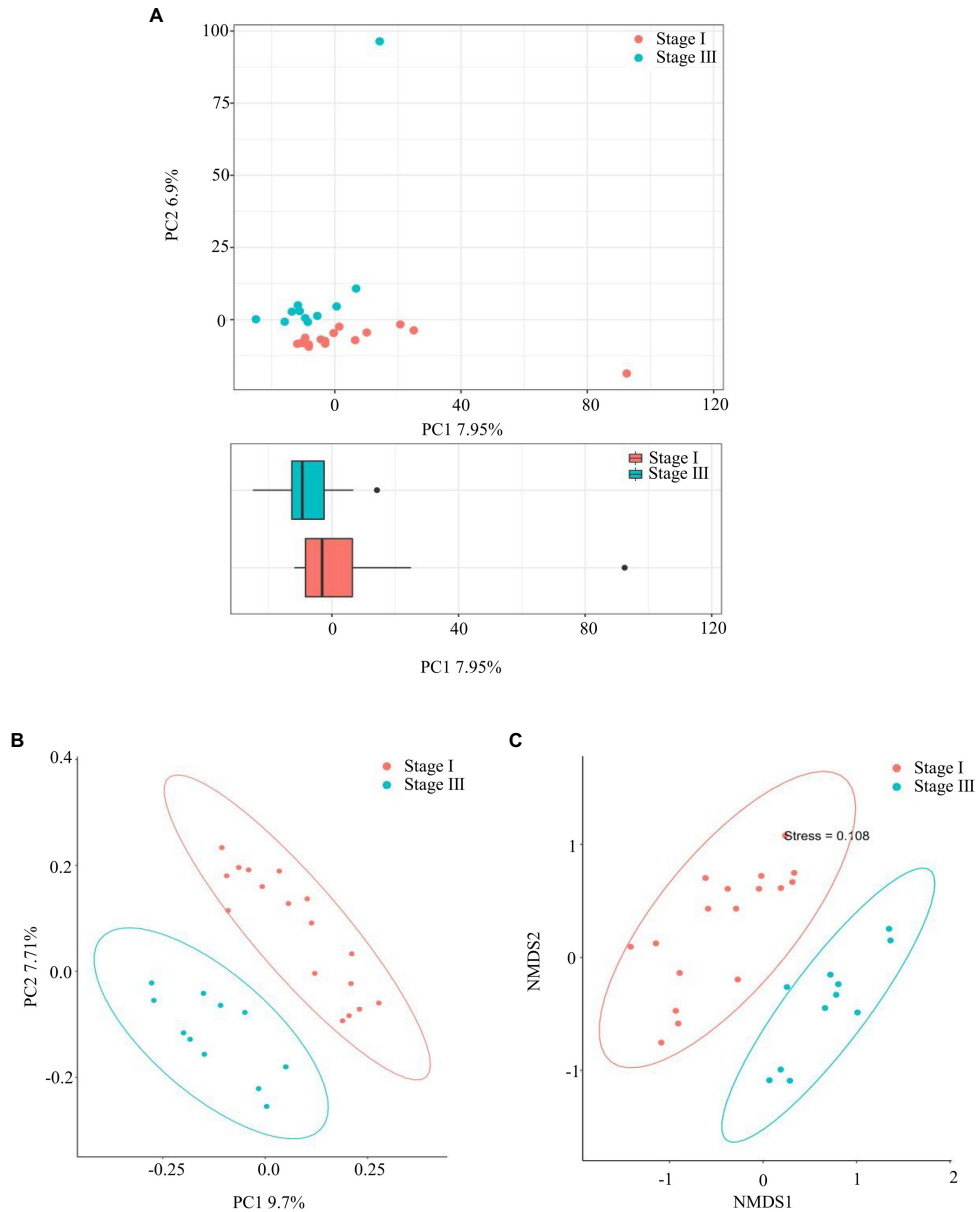
The β-diversity analysis. **(A)** Principal component analysis (PCA), **(B)** Principal coordinate analysis (PCoA) based on the Binary Jaccard distance, and **(C)** non-metric multidimensional scale based on the unweighted UniFrac distance, respectively, were used to analyze the similarity between microbial communities.

**Figure 4:**
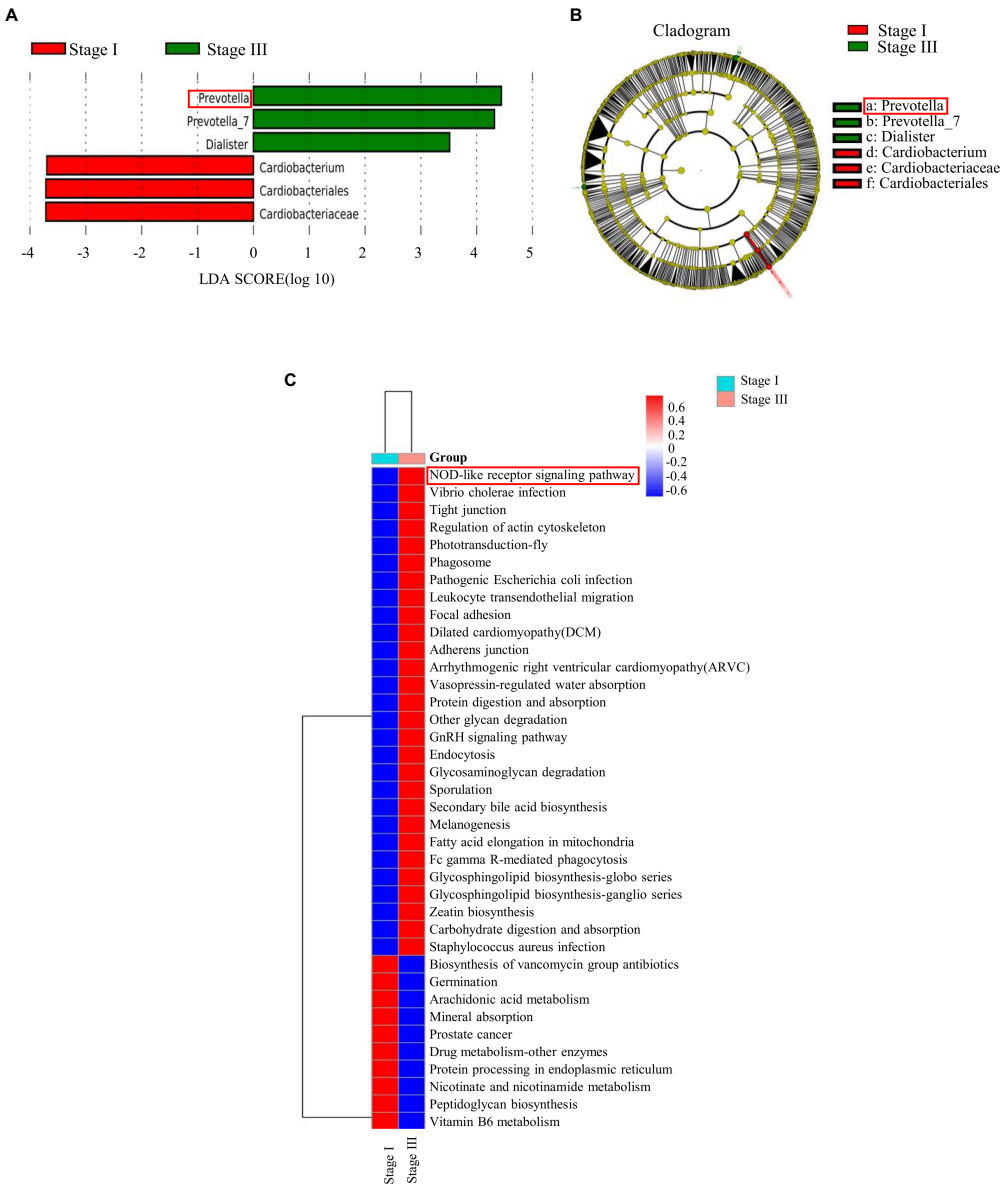
The linear discriminant analysis effect size (LEfSe) results of oral microbiomes in different groups, and pathway enrichment analysis based on the Kyoto Encyclopedia of Genes and Genomes (KEGG). **(A)** Histogram of the comparison of linear discriminant analysis (LDA) score analysis between stage I and stage III samples. Samples of stage I periodontitis-enriched taxa are indicated with a positive LDA score (green), while taxa enriched in samples of stage III periodontitis have a negative score (red). **(B)** Taxonomic cladogram obtained from linear discriminant analysis effect size analysis. The cladogram reports the taxa (highlighted by small circles and by shading; red indicates samples of stage I periodontitis, green indicates samples of stage III periodontitis, and yellow indicates non-significant results) showing different abundance values. **(C)** The differences between all samples of stage I and stage III periodontitis were clustered into a heatmap at the L3 level of KEGG.

The α-diversity was evaluated by the Chao1, Good’s coverage, Observed Species, PD whole-tree, Shannon, and Simpson indices (Figure 2). The Chao1 index, PD whole tree values, and observed species were significantly higher in young male with stage III periodontitis (*p* < 0.05; Figures 2A–C). The stage I group had a higher Good’s coverage value compared to the stage III group (*p* < 0.05; Figure 2D). There were no significant differences in the Shannon and Simpson indices between the two groups (*p* < 0.05; Figures 2E,F). The β-diversity reflects the variation in bacterial community composition between different groups. We assessed differences in oral microbial diversity in young males with stage I and stage III periodontitis using PCA, PCoA, and NMDS analyses (Figure 3). The PCA plot indicated the degree of spatial distance between the two groups (Figure 3A). PCoA based on the binary Jaccard distance and NMDS based on the unweighted UniFrac distance, respectively, revealed a significant difference in the microbial communities between the two groups (Figures 3B,C). LEfSe was conducted to identify differences in bacterial communities between the young males with stage I and stage III periodontitis. The composition differences between the two groups were evaluated at the phylum and genus levels (Figures 4A,B). Combined with the linear discriminant analysis (LDA) score and taxonomic cladogram from the LEfSe analysis, we found that the genera *Prevotella*, *Prevotella 7*, and *Dialister* were enriched in male with stage III periodontitis, while the order *Cardiobacteriales*, its family *Cardiobacteriaceae*, and its genus *Cardiobacterium* were enriched in the male with stage I periodontitis.

### Nod-like receptor signaling pathway was enriched in young males with stage III periodontitis

Abundance of functional pathways in young males with stage I and stage III periodontitis was predicted by PICRUSt and QIIME2. There was a significant difference in enriched pathways between the two groups at different levels (Figure 4C). The predicted KEGG pathway at the L1 level showed that the altered abundance of the microbiome primarily involves pathways associated with cellular processes, genetic information processing, and environmental information processing (Supplementary Figure S2A). The results at the L2 level indicated that the pathways related to the immune system, digestive system, and cell communication were enriched in young males with stage III periodontitis (Supplementary Figure S2B). At the L3 level of KEGG analysis, activity of the NOD-like receptor signaling pathway was found to be significantly increased in male with stage III periodontitis (Figure 4C).

Through the bioinformatics analysis, it was found that *Prevotella* spp. and the NOD-like receptor signaling pathway were enriched in young males with stage III periodontitis. Subsequently, based on the HOMD database (https://homd.org/) and literatures, we selected *P*. *intermedia* from *Prevotella* spp. to verify whether it can promote periodontitis progression by regulating the NOD-like receptor signaling pathway.

### NOD-IN-1 reduced *P. intermedia* LPS-induced Tnf-α expression and promoted *P. intermedia* LPS-induced Il-6 expression

**Figure 5:**
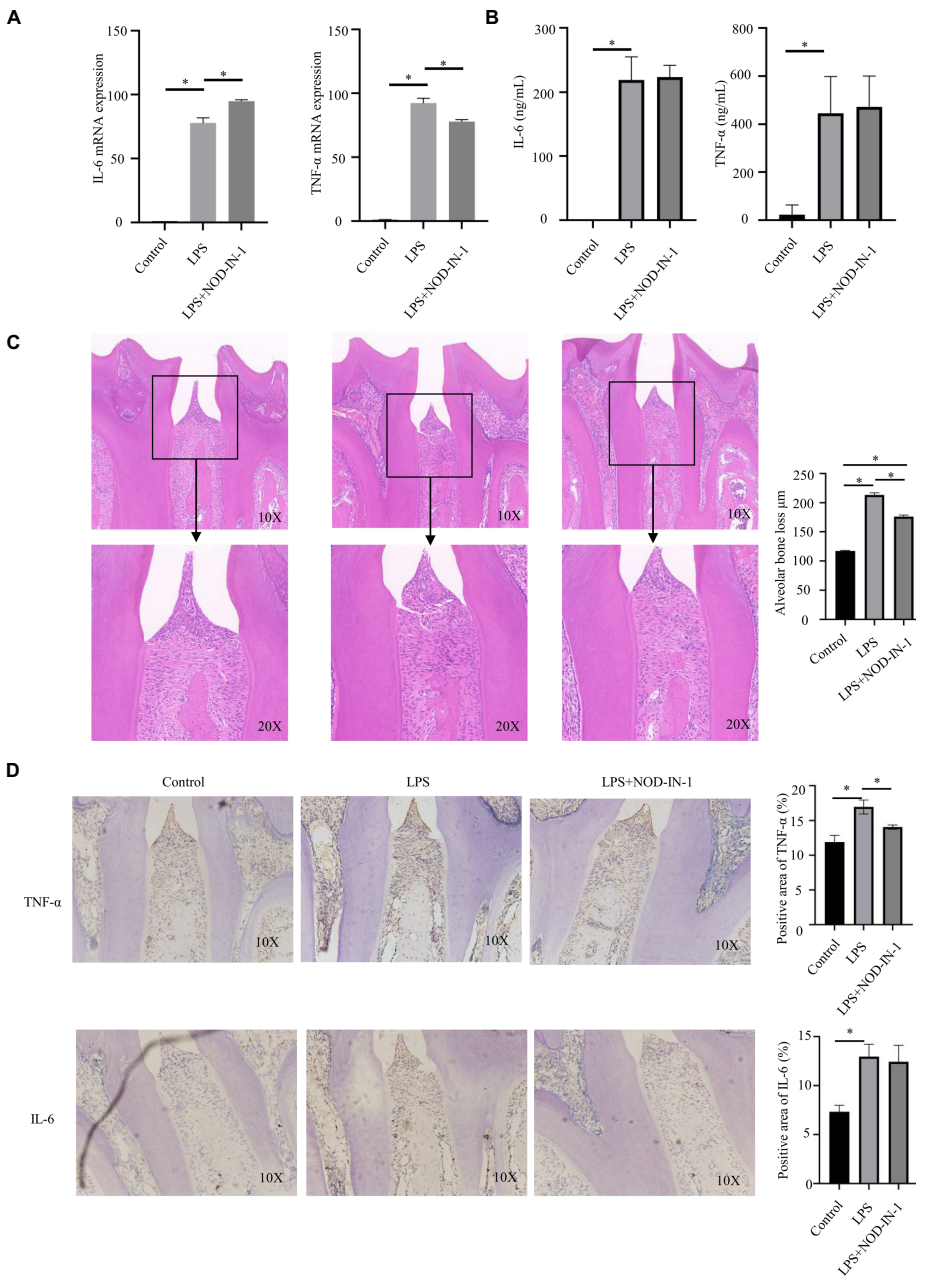
Effect of NOD-IN-1 on *P. intermedia* LPS-induced periodiontitis inflammation *in vitro* and *in vivo*. **(A)** Cells were treated with *P*. *intermedia* LPS (10 μg/mL) in the absence or presence of NOD-IN-1 for 24 h; then, real-time polymerase chain reaction was carried out for the measurement of *Il-6*, and *Tnf-α*mRNA expression. The results are presented using mean ± standard deviation values of 3 independent experiments. *p < 0.05. **(B)** Enzyme-linked immunosorbent assay results showing the levels of IL-6, and TNF-α in RAW 264.7 cells treated with *P*. *intermedia* LPS in the presence or absence of NOD-IN-1. **(C)** Representative hematoxylin and eosin images of periodontium after *P*. *intermedia* lipopolysaccharide injection with or without NOD-IN-1 application and quantitative measurement of attachment loss. **(D)** Representative immunohistostaining of TNF-α and IL-6 in the periodontium and the quantitative measurement of their expression.

Immunohistochemical results showed that NOD expression was significantly increased in the *P*. *intermedia* LPS injection group (Supplementary Figure S3). Stimulation with *P*. *intermedia* LPS brought about a significant increase in Il-6 [(*p* < 0.05; Figure 5A) and Tnf-α (*p* < 0.05; Figure 5A] mRNA expression compared to the control group. NOD-IN-1 demonstrated significant inhibitory effects on the expression of Tnf-α (*p* < 0.05; Figure 5A) and increased the Il-6 (*p* < 0.05; Figure 5A) mRNA level. The release of IL-6 and TNF-α of cells treated with *P. intermedia* LPS was significantly increased [(*p* < 0.05; Figure 5B]. After application of NOD-IN-1, the secretion of both IL-6 and TNF-α was slightly increased, but not in a statistically significant manner compared to in the LPS group (*p* > 0.05; Figure 5B).

### NOD-IN-1 inhibited inflammation infiltration in periodontitis induced by *P. intermedia* LPS

The body weights of animals did not significantly change during the experiment (Supplementary Figure S4). HE staining revealed marked increases in inflammatory cell infiltration in the gingival tissues between the first and second molars in the LPS group, with loosening of collagen fibers and a loss of their compactness (Figure 5C). A small amount of connective tissue attachment loss was also observed in the NOD-IN-1 group (Figure 5C). Additionally, the immunohistochemistry results of TNF-α and IL-6 also reflected the inflammatory response (Figure 5D); notably, the expressions of TNF-α and IL-6 were increased in the LPS group, while NOD-IN-1 pre-treatment reduced TNF-α and IL-6 upregulation induced by LPS (Figure 5D).

## Discussion

Periodontitis is a highly prevalent chronic inflammatory disease. Pathologically, it is characterized by the infiltration of inflammatory cells in the periodontal tissue, the formation of deep periodontal pockets, and the resorption of alveolar bone (Pihlstrom et al., 2005). Plaque bacteria and their products are essential initiating factors for periodontitis and directly and indirectly participate in the whole process of this disease (Lunar Silva and Cascales, 2021). A specific group of bacteria defined as “the red complex,” which includes *P*. *gingivalis*, *B*. *forsythus*, and *T*. *denticola*, is strongly related to the pathology of periodontitis (Jiang et al., 2021), while bacteria in “the orange complex,” including *P*. *intermedia*, *P*. *nigrescens*, *P*. *micros*, and *F*. *nicleatum* (Miranda et al., 2019). When the periodontium is healthy, the number of oral bacteria is low and mostly include aerobic or facultative Gram-positive streptococci and actinomycetes (Aas et al., 2005). As periodontitis progresses, however, the number of subgingival plaque bacteria increases significantly, and the relative abundance of Gram-negative anaerobic bacteria rises (Pianeta et al., 2021). This change in microbial community composition is sufficient to alter host microbial crosstalk, leading to damaging inflammation and bone loss (Hajishengallis, 2014).

Methods to detect the species and abundance of oral microorganisms include culturing (Pianeta et al., 2021), checkerboard DNA–DNA hybridization (Haffajee et al., 2009), and 16S rRNA sequencing (Zhao et al., 2021a). 16S rRNA sequencing mainly evaluates the species composition of a bacterial community, the evolutionary relationship between species, and the diversity of the community (Zhao et al., 2021a). The evaluation indicators primarily include α-diversity, β-diversity, and LEfSe analysis (Zhao et al., 2021a,b). In our study, we found that values of α-diversity indices (Chao1, Observed Species, and PD whole-tree) of young males with stage III periodontitis were greater than those of young males with stage I periodontitis, indicating that the former group had a greater richness of oral bacteria. This trend may be due to the increase in the number of Gram-negative bacteria as periodontitis progresses (Ansiliero et al., 2021). Our results are consistent with those of other studies (Genco et al., 2019; Relvas et al., 2021). Relvas et al. (2021) revealed that increased α-diversity was observed in stage 2/3 periodontitis compared to stage 1 periodontitis. Genco et al. reported that old women aged 53–81 with severe periodontitis had greater α-diversity compared to those with none/mild or moderate periodontitis (Genco et al., 2019). As periodontitis progresses, the composition of the subgingival oral community shifts. Such alterations in bacteria composition may take time over the course of periodontitis to be reflected by β-diversity. Relvas et al. (Relvas et al., 2021) also revealed that there was no significant difference in β-diversity when similar degrees of periodontitis were compared, whereas β-diversity showed differences when healthy (stage 0) patients were compared to those with severe periodontitis (stage 2/3). Tomšič et al. (Tomšič et al., 2021) found no differences in detection frequency and counts of most major periodontal pathogens between patients with stage III and IV periodontitis, respectively. In our study, the β-diversity results suggested that the oral flora of young males with stage I and III periodontitis differed.

Our results suggested that *Cardiobacterium* is a potential marker of the oral microbiota in young males with stage I periodontitis. *Cardiobacterium* is a genus of Gram-negative bacteria that often colonizes the oral cavity. Colombo et al. (Colombo et al., 2009) revealed that *Cardiobacterium* is significantly more prevalent in periodontally healthy patients than periodontitis patients. Moreover, *Cardiobacterium* has a positive association with periodontal therapy success, as it was found at greater frequencies in sites with good therapeutic effects (Colombo et al., 2012). In our study, at the genus level, increased *Prevotella*, *Prevotella_7*, and *Dialister* abundance in the oral microbiota of young males caused periodontal destruction. *Dialister*, a genus of anaerobic Gram-negative bacteria, is related to the occurrence and development of oral diseases and mainly participates in apical periodontitis lesions (Doan et al., 2000; Rôças et al., 2011). Contreras et al. (Contreras et al., 2000) found that *Dialister pneumosintes* was detectable in 83% of patients with severe periodontitis. *Dialister pneumosintes* had significant correlation with pocket depth, attachment loss, and disease-active periodontitis (Slots et al., 2002; Ferraro et al., 2007). *Prevotella* is a genus of Gram-negative bacteria and is one of the most important genera of bacteria in the oral cavity (Segata et al., 2012; Tett et al., 2021). Among the *Prevotella* species (including *Prevotella* and *Prevotella_7*), *P*. *intermedia* is part of the “orange complex” and is associated with chronic periodontitis, aggressive periodontitis, and gingivitis (Könönen and Müller, 2014; Gambin et al., 2021). As for another *Prevotella soecies P*. *nigrescens*, it was observed in in the subgingival plaque of periodontitis patients and induced host IL-1β production in dendritic cells (Jang et al., 2021). In this study, *P*. *intermedia* LPS induced the expression of both IL-6 and TNF-α in RAW264.7 cells, and gingival injection of LPS caused peritodontal inflammation *in vivo*. Consistently, LPS increases the release of inflammatory factors from macrophages (Choi et al., 2015; Choe et al., 2019).

The NOD-like receptor signaling pathway is the most enriched pathway in young males with stage III periodontitis. The NLR family is an intracellular sensor family and participates in sensing a variety of bacterial pathogens (Platnich and Muruve, 2019). NOD1 and NOD2 are two types of NLRs and are involved in the recognition of periodontal pathogens and the process of periodontitis (Okugawa et al., 2010; Marchesan et al., 2016). It has been reported that NOD1 and NOD2 can respond to stimulation by *P*. *gingivali* (Liu et al., 2014). It is controversial whether NOD1/NOD2 plays a pro-inflammatory or anti-inflammatory role in periodontitis. It was reported that the deficiency of NOD1 exacerbated bone resorption (Chaves de Souza et al., 2016) while another study revealed that mice lacking NOD1 showed reduced bone resorption and impaired recruitment of neutrophils in gingival tissue and alveolar bone (Jiao et al., 2013). Souza et al. (2016) observed that NOD2-knockout mice had significantly reduced bone resorption and osteoclastogenesis, but their severity of inflammation was unaffected. Another study found that NOD2 promoted *P*. *gingivalis*–induced bone resorption (Prates et al., 2014). In contrast, Jiao et al. found in a model of periodontitis induced by silk ligation that alveolar bone absorption and neutrophil infiltration were not altered in NOD2-knockout mice (Jiao et al., 2013). Due to the importance of NOD-like receptor signaling in periodontitis, we subsequently selected an inhibitor of this pathway to explore the effects of P. intermedia LPS on periodontitis *in vivo* and *in vitro*. NOD-IN-1 is a potent mixed inhibitor of NOD1 and NOD2; at concentrations below 10 μM, it exhibits balanced inhibitory activity on both targets (Keček Plešec et al., 2016). In our study, the inhibitory activity of NOD-IN-1 in the NLR pathway was verified.

After application of NOD-IN-1, Tnf-α mRNA expression induced by *P*. *intermedia* LPS was markedly suppressed, while Il-6 mRNA expression further increased *in vitro*. The expressions of IL-6 and TNF-α in periodontal tissue decreased *in vivo* after intraperitoneal injection of NOD-IN-1. NOD-IN-1 regulated both mRNA and protein levels of TNF-α and IL-6. TNF-α showed consistent changes *in vitro* and *in vivo*, which may be due to the fact that TNF-α production is more sensitive to external intervention and the existence of translational activation (Di Marco et al., 2001) Previous study noted that LPS is the only stimulator clearly shown to activate the translational effect of TNF-α in primary monocytes and macrophages (Schook et al., 1994). The reasons for the inconsistent changes in the levels of IL-6 mRNA and protein secretion *in vitro* and *in vivo* may be that IL-6 does not have such a strong translation effect, making it less sensitive to stimuli. Some scholars reported that the mRNA and biological activity levels of TNF-α are strongly inhibited under the same stimulation, while IL-6 production is unaffected (Schook et al., 1994). NOD-IN-1 was applied *in vivo* for a longer time (13 times in 26 days), while the detection of intracellular mRNA was carried out just 1 day after LPS and NOD-IN-1 intervention. The hyposensitivity of IL-6 may lead to a temporary increase in Il-6 mRNA.

## Conclusion

The composition of the oral bacteria in young males varied according to the stage of periodontitis. The species richness of oral microtia was greater in young males with stage III periodontitis than those with stage I periodontitis. *Prevotella* was the dominant bacteria in young males with stage III periodontitis, and inhibition of the NOD-like receptor signaling pathway can decrease the periodontal inflammation induced by *P*. *intermedia*.

## Data availability statement

The datasets presented in this study can be found in online repositories. Raw reads have been deposited at NCBI under the BioProject accession number PRJNA893253.

## Ethics statement

The studies involving human participants were reviewed and approved by the Ethics Committee of the Second Xiangya Hospital of Central South University. The patients/participants provided their written informed consent to participate in this study. The animal study was reviewed and approved by the Experimental Animal Ethics Committee of the Second Xiangya Hospital.

## Author contributions

YZ and QY contributed to conception and design, acquisition, analysis, and interpretation, drafted manuscript, made important contributions to the revision of manuscript and gave final approval; YF and YG contributed to conception and design, acquisition, analysis, and interpretation, drafted manuscript, critically revised manuscript and gave final approval; YF, YC, LT, ZO, and JZ contributed to acquisition and interpretation, critically revised manuscript, and gave final approval; JH and NC contributed to analysis, critically revised manuscript and gave final approval; MD and XS contributed to acquisition, critically revised manuscript and gave final approval. All authors contributed to the article and approved the submitted version.

## Funding

This study was supported by the National Natural Science Foundation of China (81800788 and 81773339), Science and Technology Department of Hunan Province, China (2017WK2041 and 2018SK52511), Scientific Research Project of Hunan Provincial Health Commission (202208043514), Hunan Provincial Natural Science Foundation of China (2022JJ30062), Natural Science Foundation of Changsha City (kq2202403 and kq2202412), Fund for the Xiangya Clinical Medicine Database of Central South University (2014-ZDYZ-1-16), Education and Teaching Reform Research Project of Central South University (2020jy165-3), Research Project on Postgraduate Education and Teaching Reform of Central South University (2021JGB072), Open Sharing Fund for the Large-scale Instruments and Equipment of Central South University and Fundamental Research Funds for the Central Universities of Central South University.

## Conflict of interest

The authors declare no potential conflicts of interest with respect to the authorship and/or publication of this article.

## Publisher’s note

All claims expressed in this article are solely those of the authors and do not necessarily represent those of their affiliated organizations, or those of the publisher, the editors and the reviewers. Any product that may be evaluated in this article, or claim that may be made by its manufacturer, is not guaranteed or endorsed by the publisher.
