# Supplementary material for: Prevotella genus and its related NOD-like receptor signaling pathway in young males with stage III periodontitis
Source: Front Microbiol. 2022 Dec 8;13:1049525. doi: 10.3389/fmicb.2022.1049525 (PMC9772451; doi:10.3389/fmicb.2022.1049525)
Supplement: Supplementary file 1 [file Data_Sheet_1.docx]

Supplementary Material

# Supplementary Figures and Tables

## Supplementary Figure


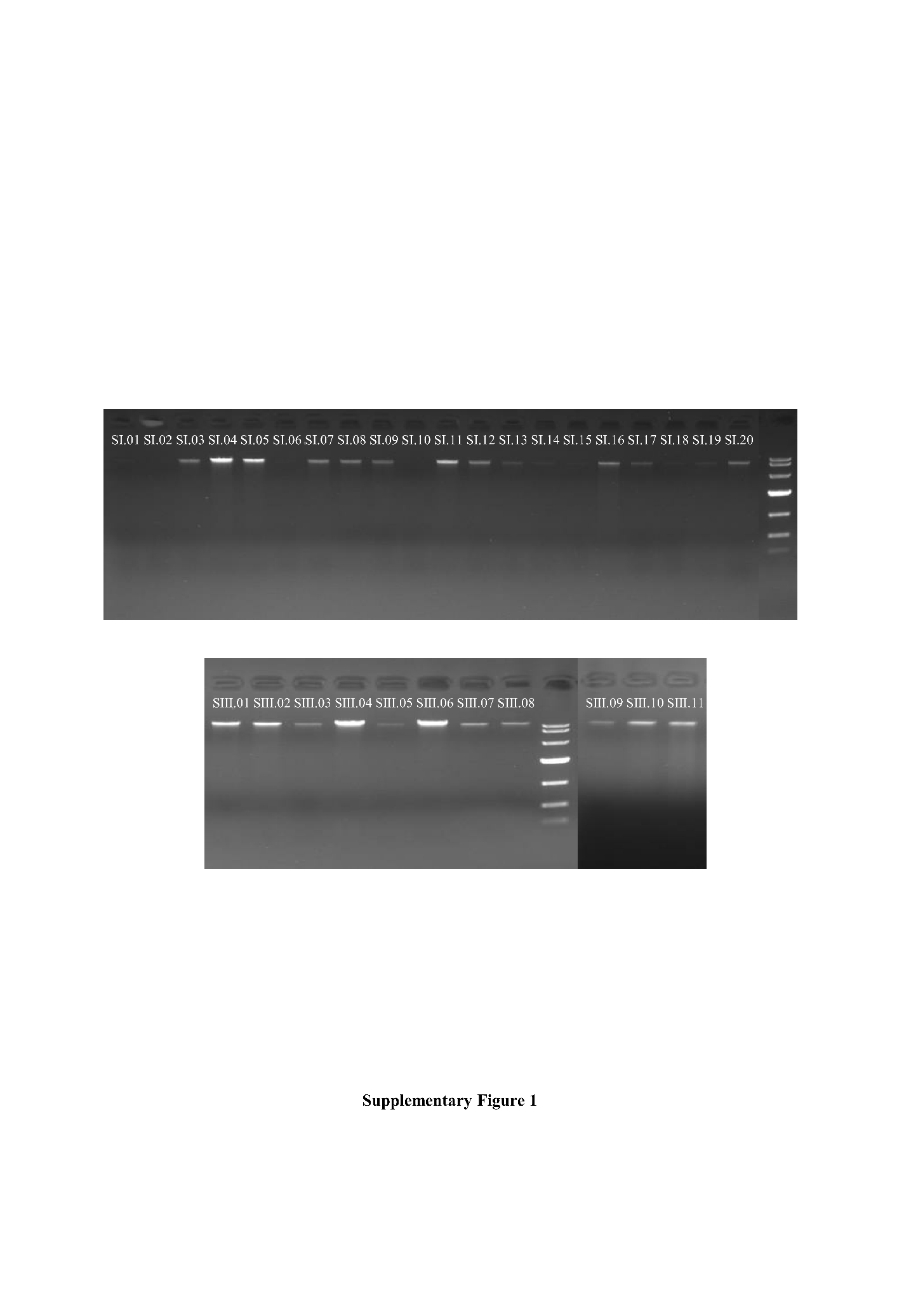


**Supplementary Figure 1.** Agarose electropherogram of DNA from Subgingival plaque samples.
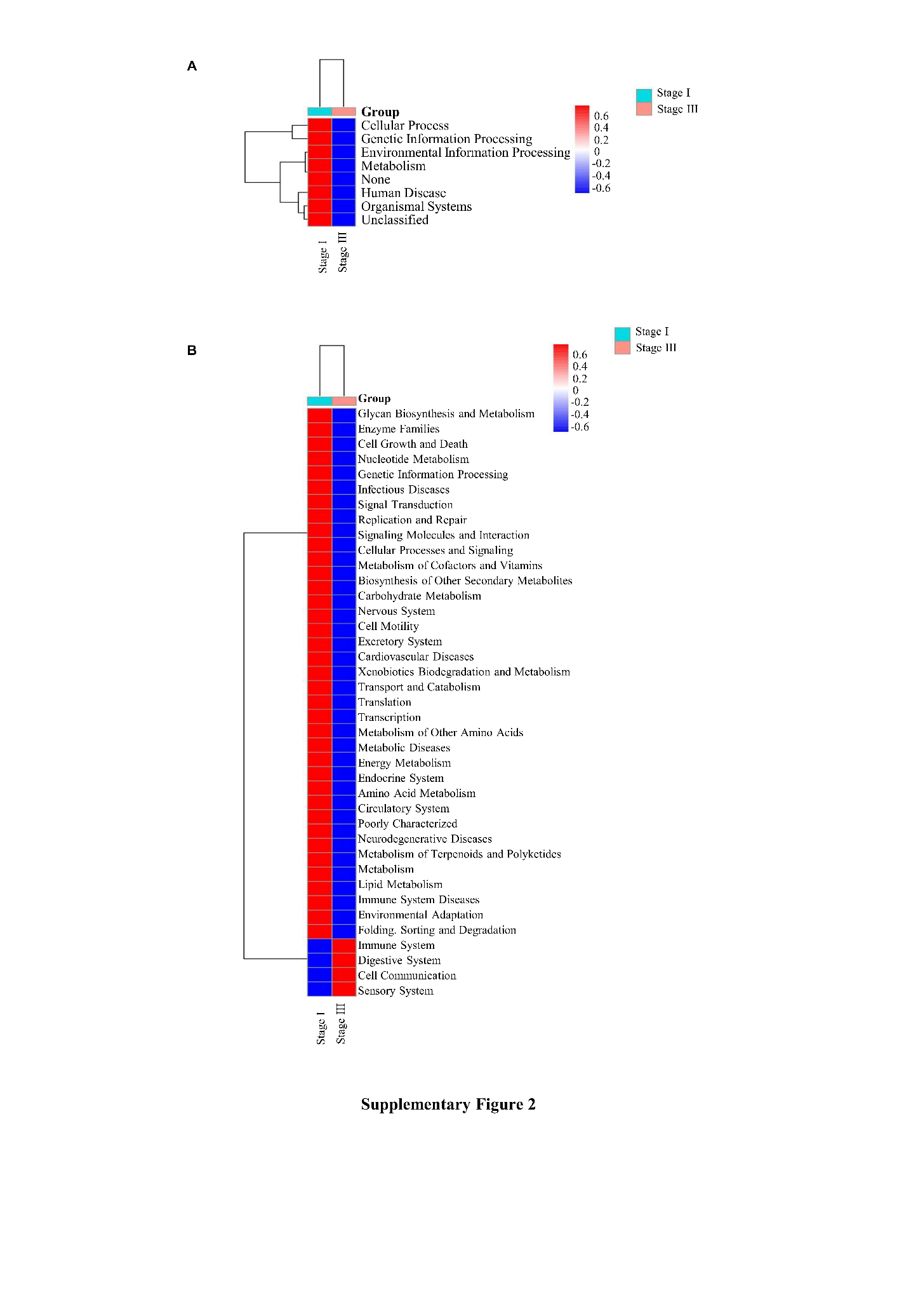


**Supplementary Figure 2.** Pathway enrichment analysis based on KEGG. At the L1(a) and L2(b) levels of KEGG, the differences of all samples in Grade Ⅰ and Grade Ⅲ were clustered into heat map.


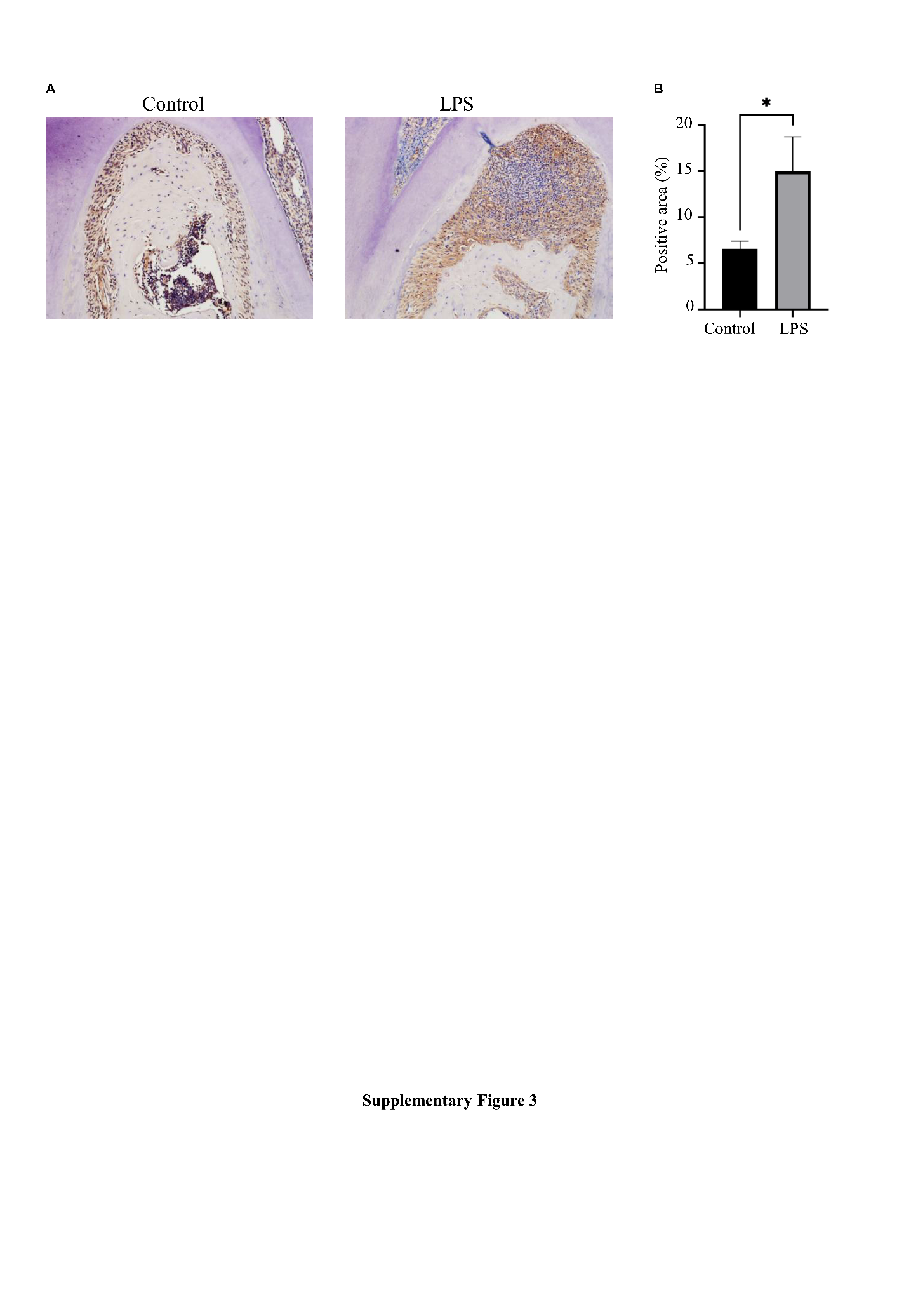


**Supplementary Figure 3.** Representative immunohistostaining of NOD in the periodontium of the Control group and *P. intermedia* lipopolysaccharide injection group.


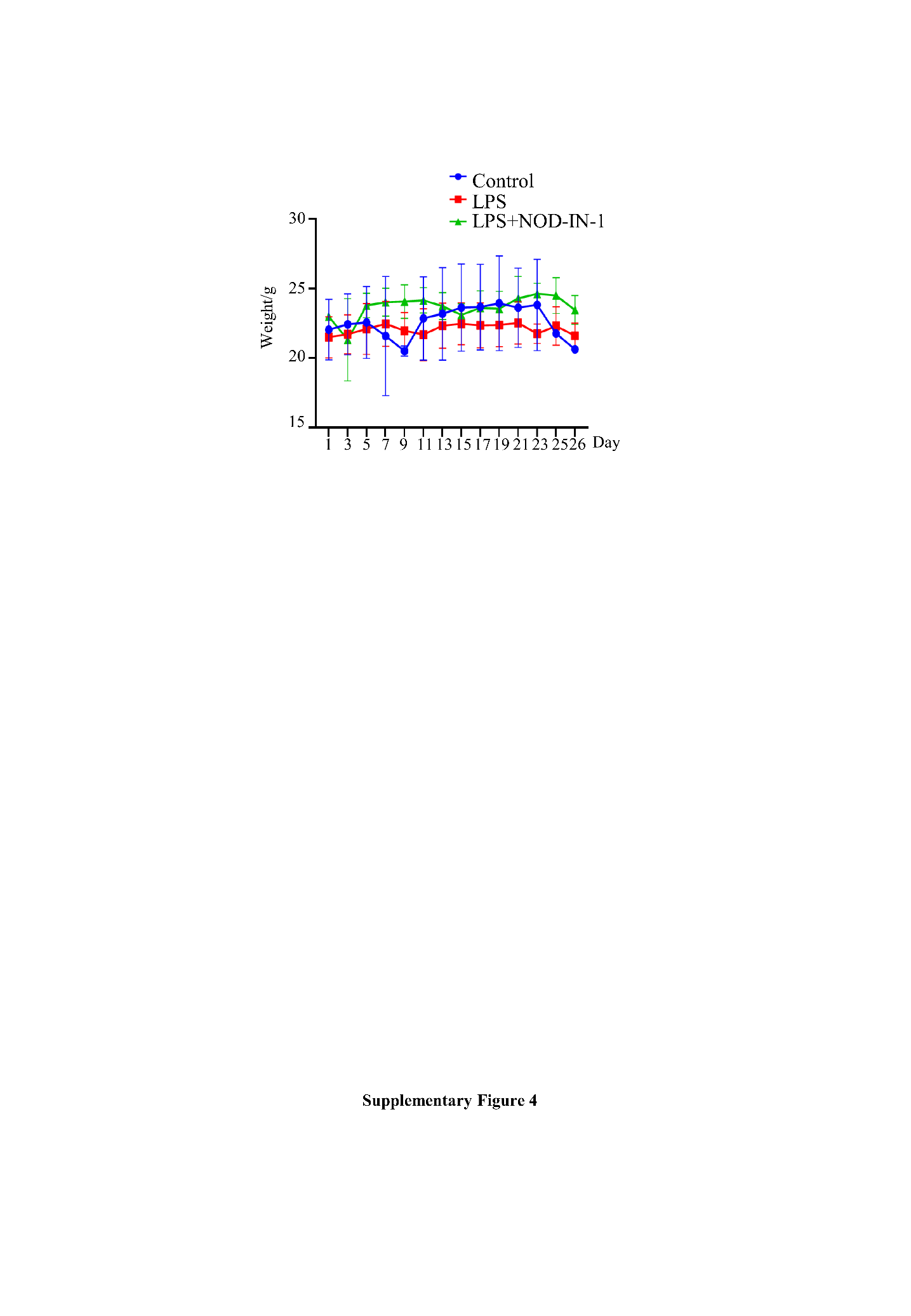


**Supplementary Figure 4.** The changes of body weight in each group during gingival injection.

## Supplementary Tables

Table S1 The differential abundance of genera in the Grade Ⅰ and Ⅲ group.

| Genus name | Grade Ⅰ  mean abundance | Grade Ⅲ  mean abundance | Fold change | p value |
| --- | --- | --- | --- | --- |
| Prevotella | 0.0476 | 0.1007 | 2.1145 | 0.0170 |
| Prevotella_7 | 0.0201 | 0.0567 | 2.8245 | 0.0330 |
| Dialister | 0.0026 | 0.0085 | 3.2922 | 0.0390 |
| Cardiobacterium | 0.0161 | 0.0066 | 2.4423 | 0.0200 |
| Actinomyces | 0.0068 | 0.0049 | 1.3946 | 0.0290 |
| Bergeyella | 0.0044 | 0.0026 | 1.6835 | 0.0150 |
